# Weather fluctuations: predictive factors in the prevalence of acute coronary syndrome

**DOI:** 10.15171/hpp.2019.17

**Published:** 2019-05-25

**Authors:** Hamid Sharif Nia, Yiong Huak Chan, Erika Sivarajan Froelicher, Saeed Pahlevan Sharif, Ameneh Yaghoobzadeh, Azar Jafari, Amir Hossein Goudarzian, Roghiyeh Pourkia, Ali Akbar Haghdoost, Farhad Arefinia, Roghieh Nazari

**Affiliations:** ^1^Department of Medical-Surgical Nursing, School of Nursing and Midwifery Amol, Mazandaran University of Medical Sciences, Sari, Iran; ^2^Biostatistics Unit, Yong Loo Lin School of Medicine, National University Health System, Singapore; ^3^Department of Physiological Nursing, Department of Epidemiology & Biostatistics, University of California San Francisco, California, USA; ^4^Taylor’s Business School, Taylor’s University Malaysia, Subang Jaya, Malaysia; ^5^Department of Nursing, Tehran University of Medical Sciences, Tehran, Iran; ^6^Department of Nursing, Mazandaran University of Medical Sciences, Sari, Iran; ^7^Faculty of Nursing, Student Research Committee, Mazandaran University of Medical Sciences, Sari, Iran; ^8^Department of Cardiology, Cardiovascular Research Center, Babol University of Medical Sciences, Babol, Iran; ^9^Modeling in Health Research Center, Institute for Futures Studies in Health, Kerman University of Medical Sciences, Kerman, Iran; ^10^School of Nursing and Midwifery Amol, Mazandaran University of Medical Sciences, Sari, Iran

**Keywords:** Meteorological parameters, Seasonal changes, Acute coronary syndrome, Prevalence, Iran

## Abstract

**Background:** Meteorological parameters and seasonal changes can play an important role in the occurrence of acute coronary syndrome (ACS). However, there is almost no evidence on a national level to suggest the associations between these variables and ACS in Iran. We aim to identify the meteorological parameters and seasonal changes in relationship to ACS.

**Methods:** This retrospective cross-sectional study was conducted between 03/19/2015 to 03/18/2016 and used documents and records of patients with ACS in Mazandaran ProvinceHeart Center, Iran. The following definitive diagnostic criteria for ACS were used: (1) existence of cardiac enzymes (CK or CK-MB) above the normal range; (2) Greater than 1 mm ST-segment elevation or depression; (3) abnormal Q waves; and (4) manifestation of troponin enzyme in the blood. Data were collected daily, such as temperature (Celsius) changes, wind speed and its direction, rainfall, daily evaporation rate; number of sunny days, and relative humidity were provided by the Meteorological Organization of Iran.

**Results: ** A sample of 2,054 patients with ACS were recruited. The results indicated the highest ACS events from March to May. Generally, wind speed (18 PM) [IRR = 1.051 (95% CI: 1.019 to1.083), P=0.001], daily evaporation [IRR = 1.039 (95% CI: 1.003 to 1.077), P=0.032], daily maximum (P<0.001) and minimum (P=0.003) relative humidity was positively correlated withACS events. Also, negatively correlated variables were daily relative humidity (18 PM) [IRR =0.985 (95% CI: 0.978 to 0.992), P<0.001], and daily minimum temperature [IRR = 0.942 (95%CI: 0.927 to 0.958), P<0.001].

**Conclusion:** Climate changes were found to be significantly associated with ACS; especially from cold weather to hot weather in March, April and May. Further research is needed to fully understand the specific conditions and cold exposures.

## Introduction

Acute coronary syndrome (ACS) is one of the most common health problems in the world, which can increase mental health problems, result in considerable disability, increase morbidity, and mortality.^1,2^ Studies during several decades have focused on different pathophysiological mechanisms and predisposing factors for the incidence of ACS.^3-5^ One of these studies focused on the effects of weather conditions on incidence of ACS that has been discussed in different areas of the world for more than 50 years.^6^ A number of investigators found that meteorological parameters and seasonal changes can play an important role in the occurrence of ACS.^7,8^ The role of temperature is both direct and fast. Time series studies have revealed that there is a minimum lag time of about 24 hours between decrease in temperature and increase in mortality.^9^ The study reported that approximately 4% of ACS onsets are associated with different kinds of meteorological parameters.^10^ In another study, it was found that weather conditions like atmospheric air temperature, humidity, wind speed, and wind pressure effects the incidence of ACS.^11-13^ Although several studies suggest that ACS occurs most often in the winter months,^14-17^ other studies report that the occurrence rate of ACS increases in spring.^6,18^ Yet another study shows that ACS occurs in summer when the temperature and humidity are high and atmospheric pressure is low.^19^ It has also been shown that there is an association between temperature, high relative humidity, and strong winds and ACS.^9^ It seems that physiologic stressors such as sympathetic activation, hypercoagulability and infection in cold weather condition (such as influenza epidemics and air pollution) are linked to the incidence of ACS.^20^ Additionally, hemodynamic changes are exacerbated during winter, as are elevated immune reactions, uncontrolled hypertension, immobility, and risk for respiratory infections,^21^ that can affect the incidence of ACS. Some studies state that cold temperature effects platelet numbers, arterial blood pressure, and thrombus formation.^19^ Also, variation of incidence of ACS in different seasons is attributed to variability in ultraviolet-B exposure during the seasons and vitamin D deficiency that may increase the cardiovascular risk.^22^ Nevertheless, a 1°C decrease in temperature caused a 1%–2% rise in the number of deaths.^9^

Although‏ numerous‏ studies‏ have‏ been‏ conducted‏ on‏ the impact of climate variables and seasonal changes on the risk of ACS in different areas of the world; many of these studies lack national level data. However, there is almost no evidence to suggest an association between climate variables and the risk of ACS in Iran. So, due to the importance of reducing the incidence of ACS and its effects on quality of life, it is important to study the impact of meteorological parameters and seasonal changes on the incidence of ACS in a region with different weather conditions and great geographic diversity. In other words, a better understanding of the seasonal changes may provide novel pathways to prevent ACS.^23^ Therefore, the present study aims to determine the relationship between meteorological parameters with the incidence of ACS.

## Materials and Methods

This retrospective cross-sectional study design was used. This study is based on medical records of the Heart Center of Mazandaran province, Iran. This center provides the most comprehensive data in northern Iran on all patients with a diagnosis of ACS.

### Setting

The Mazandaran Province Heart Center, Iran located at the following coordinates (36.369 N, 52.270 W) was chosen because it offers the most complete data in Iran about patients diagnosed with ACS.^24^ Census sampling method was used. Existing date was used between 03/19/2015 to 03/18/2016. Sari (the capital of Mazandaran) is a north city of Iran which has mild weather. Based on the newest census in 2016, it contains the 505 000 inhabitants. The center consists of five CCU wards, one ICU, and one emergency ward. Registered data of the ACS patients surveyed are used from these units.

### Study population

The current study was carried out among all patients referred to the hospital with symptoms of ACS. The cardiologist, two nurses, a statistician, and an epidemiologist (who extracted and recorded the needed information using a data collection guide) formed the research team. The final diagnosis of ACS was verified by the cardiologist. The following were considered as the definitive diagnostic criteria were: (1) existence of cardiac enzymes (CK or CK-MB) above the normal range; (2) ST-segment elevation or depression of more than 1; (3) abnormal Q waves; and (4) manifestation of Troponin enzyme in the blood.^2^

Two nurses invited patients to participate in the study after the ethical approval of the study had been obtained from the Mazandaran University of Medical Sciences.

### Measurements 

The following variables were abstracted: gender, the day, month, year and time of hospital admission. Also, weather variables were included daily temperature (Celsius) changes (minimum, maximum, and average), wind speed (meters per second) and its direction, rainfall (day), daily evaporation rate (mm), number of sunny days, and relative humidity (percent) between March 2015 to March 2016 were provided by the Meteorological Organization of Iran. Iran’s four climate seasons are: spring (April to June), summer (July to September), autumn (September to December) and winter (January to March).

### Statistical analysis

All analyses were performed using SPSS 24.0 (SPSS 24.0, Inc., Chicago, Illinois, USA) with statistical significance set at α = 0.05. Mean (SD) were presented for numeric normal variables, median (range) for numeric non-normal variables and frequency (%) for categorical variables. General Linear Model was performed to compare the number of ACS events across months adjusting for age, gender and meteorological variables with Bonferroni correction for pairwise comparisons. A negative binomial regression model accounting for over-dispersion was used to determine the meteorological (daily temperature (minimum, maximum, and average), wind speed and its direction, rainfall, daily evaporation rate, number of sunny days, and relative humidity) and demographical predictors on daily (defining a day from 00:00 to 23:59 hours) relative risk (RR) ACS prevalence with a 95% confidence interval, gender-subgroup analyses was also performed.

## Results

### Sample characteristics

Over the study period, a total of 2054 patients with ACS were recruited. The mean (±SD) age of the subjects was 55.6 (±13.4), median 58 and their ages ranged from 20 to 91 years old, with 49.3% being men. The ages for men were mean (±SD) 56.3 (±13.3), median 58, ranging between 20-91 and 55.0 (±13.5), 57, 20-91 for women. Table 1 shows the descriptive statistics for the meteorological variables.

### Weather fluctuations

**Figure 1:**
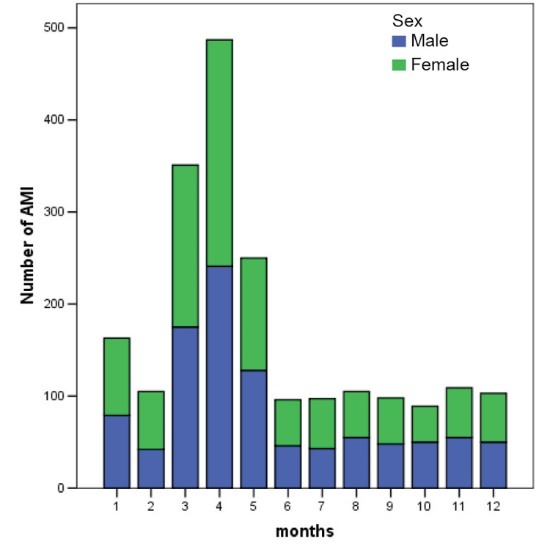
Number of AMI events by gender over month.

**Figure 2:**
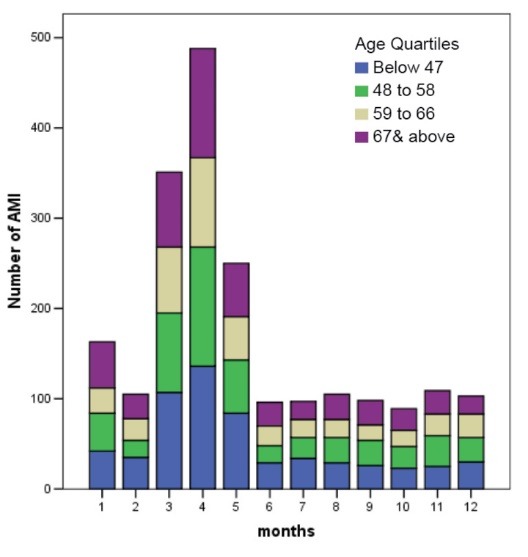
Number of AMI events by age quartiles over month.

Figures 1 and 2 show the number of ACS events by gender and by age in quartiles for each month, respectively.

Figures 1 and 2 show that the third to the fifth months had statistically higher (*P* < 0.001) occurrences of ACS events during the year. Overall the other months except for month 5 over month 1 (*P* = 0.581) adjusting for demographical and meteorological variables with Bonferroni correction (see Table 2).

### Factors associated with ACS

Table 3 shows that the variables that were positively correlated with ACS events were wind speed (18 PM), daily evaporation, maximum and minimum relative humidity. Negatively correlated variables were daily relative humidity (6 PM). The analysis by gender shows that for men positive correlates were daily evaporation and daily maximum relative humidity, trend relationship with wind speed (18 PM). For women, wind speed (18 PM), daily minimum and maximum relative humidity were positive correlates; with daily relative humidity (18 PM) and minimum daily temperature negatively correlated with ACS events.

## Discussion

The study aimed to evaluate the meteorological parameters and seasonal changes in relationship with incidence of ACS; as well as to identify gender differences. The results indicated that ACS admissions were higher March through May. Similarly March was reported as the month with the highest incidence of ACS in Germany.^25^ However, January was reported to have the highest incidence in the United Sates.^17^ Other studies found that the incidence and fatality risk of ACS were higher in the winter and spring.^4,16,18,20,26-30^ However, two additional studies reported that ACS was more common in summer.^31,32^ There are several mechanisms of climate changes that are suggested for the two pathological exogenous and endogenous responses. Lipid serum level, coagulation systems, and hormonal changes are among these features.^23^ Also, behavioral pattern variations following seasonal changes like changes in diet, physical activity, and psychosocial factors such as mood are considered as the emerging explanations for the high incidence of ACS in these seasons.^33^ Besides, variation in temperature,^34^ seasonal pattern occur such as infections like influenza epidemics,^35^ elevated concentration of fine element air pollution,^10^ seasonality phenomenon (i.e. winter depression, anxiety, sadness, social withdrawal, sleep disturbances, irritability, etc),^10^ respiratory tract infections,^36^ and reduction in the number of solar light hours^37^ are other proposed factors of the incidence of ACS. Therefore, differences in the patterns of ACS prevalence according to the time of year and changes in ambient climate within the same location may be reasonable explanations. This concept appears to be especially applicable to the regions of the world subjected to four distinct seasons and significantly different winter-to-summer weather conditions.^38^

The possible role of meteorological variables has been considered throughout the study. The present study confirmed the positive relationships between wind speed and the incidence of ACS. Goerre et al reported the same findings in their study in Switzerland.^39^ An inverse relationship was noted in the 10-year ecological study in Great Britain^40^ and a 12 years survey in Kaunas^11^ reported also negative correlations. However, another study failed to observe any significant relationships.^23^

It was investigated that relative humidity was among the factors that negatively correlated with the ACS incidences. Our findings concur with those by Abrignani et al,^23^ Messner et al,^41^ and Lee et al.^42^ one findings stated no correlations^43^ and two others reported positive relationships among these variables.^44,45^ Moreover, it seems that the presence of high air humidity may hinder swelling and also make it difficult the automatic processes of internal temperature control. Therefore, the respiratory fatigue and heart rate will be increased.^23^

Data on the role of environmental temperature are conflicting. One of the remarkable results of the present study was the negative association between daily minimum temperature and the hospital admission due to ACS; this suggests that the daily minimum temperature has a protective role in ACS. In other words, when the temperature is at lowest, it reduced ACS by 6 percent. Some studies indicated that the number of ACS are linked with the both colder and warmer temperatures.^44,46,47^ A 9-year survey (i.e. 2000-2009) in Hong Kong and Taiwan indicated that the lower mean temperature was associated with lower ACS risk on the same day.^7^ However, Kysely et al^48^ and Näyhä^49^ believe that fatality related to cold or heat is certainly not affected by hypo or hyperthermia. Also, Stewart et al reported that there is some evidence showing cold adaptation through longer exposure to the cold weather may occur. However, this approach is debatable.^38^ Also reduction in acute phase mortality, due to variations, such as earlier diagnosis of infarction, early and aggressive treatment, suitable reperfusion treatment, additional precise delineation of post ACS risk, as well as more suitable treatment of heart failure and mechanical complications after ACS are among the possible factors leading to reduction in morbidity and mortality following ACS.^50,51^ It also can be caused by indirect effects including cardiovascular disease that is exacerbated by physiological reactions of the man`s body aimed to adapt to the thermal environment.^48,49^ To the best of our knowledge, there is no study that reported a negative correlation between the minimum daily temperature and ACS incidence. Further research is needed to fully understand the individual conditions and cold exposure.

Given the fact that seasonal weather effects the prevalence, complications and outcomes of ACS, so that patients should modify their lifestyle particularly during the cold months with a diet rich in vitamins (e.g. vitamin D3), modifies activity level, suitable and warm clothes.^52^

Although the data of the current study have been extracted from the patients referred to the Sari, capital of Mazandaran city, our findings will be generalized to predict the occurrence of ACS in order to take preventive and therapeutic across the southern Caspian Sea. This happening can be due to the two causes; at first, weather characteristics as well as seasonal changes in the southern parts of the Caspian Sea follow relatively similar pattern^53^ and the next is that the residents of these areas are prone to vulnerability because of high population density.^54^

### Limitation

Similar to most studies, this study had several limitations: (1) the use of existing medical records that were collected for the purpose of diagnosis and treatment and not specifically for the purpose of this‏ research may not be ideal; (2) Over reporting, underreporting, and errors in reporting results in misclassification; (3) Lack of access to the details of all patient records (data including type of AMI, body mass index, blood pressure, past medical history, blood urea nitrogen, creatinine) precludes more detailed results; (4) On the other hand, a limitation of the present study was that we relied on central station monitoring for meteorological factors instead of measurements of exposure to environmental variables; (5) Usually in these hospitals, a wide range of patients with ACS and similar diseases are recorded in health information system. So we gathered all of them for the specified interval; (6) Another limitation to consider is that patients who died before reaching the hospital or patients that were not admitted to any hospital (outpatients) may have been excluded, thus underrepresenting the sample; (7) the possibility of having admitted patients from other provinces to this study could not be verified. Thus, caution must be exercised when interpreting the study results.

Nevertheless, several unique features of this study are the large sample size; we relied on central station monitoring for meteorological factors instead of measurements of exposure to environmental variables this provided us the dependent variables (meteorological data) data that was unbiased with regards to outcome of this study; and lastly using these data bases allowed us to answer research questions and generate new hypothesis for testing in future studies without the exorbitant cost of planning a prospective study.

### Recommendation

We recommended that more detailed studies be conducted to verify the present results by other investigators. More detailed results about the incidence of ACS regarding to seasonal changes can help us in planning and thus potentially reducing ACS. Future studies with samples from different populations and also longitudinal designs are suggested to verify the findings of this study. Importantly, this study provides useful data that can be applied to future studies. Future studies are recommended that incorporate more detailed patients information (such as type of ACS, body mass index, blood pressure, past medical history, blood urea nitrogen, creatinine), wider climate areas (such as warm and dry; cold and dry).

## Conclusion

Climate changes were found to be significantly associated with ACS. Especially from cold weather to hot weather in March, April and May. Therefore, emergency treatment service personnel should be more vigilant and fully prepared in March, April, and May for an increase in ACS patient admissions.

## Ethical approval

The study was approved (Code: IR.MAZUMS.REC.96-10232) by the Ethics Committee of Mazandaran University of Medical Sciences, Sari, Iran, pursuant to its code of ethics, including assured confidentiality of all patient information.

## Competing interests

The authors report no conflicts of interest. The authors alone are responsible for the content and writing of this article.

## Authors’ contributions

HShN, YHC, RN, and AHG were on the management committee. HSHN, ESF, AHG, AKH, RP, and AY were on the scientific committee. RP, SPSh, HSHN, AKH, FA, and ESF were responsible for data interpretation and writing the report. RP, SPSh, and AKH did the statistical analysis. HShN, AHG, AY, RN, and RP were on the writing committee. HShN, AY, ESF, YHC, FA, AKH, and SPSh reviewed and revised the manuscript. All authors reviewed the manuscript.

## Acknowledgments

This project was an inter-university collaboration and was supported by Mazandaran University of Medical Sciences, Taylor’s University and University of California San Francisco. We want to hereby extend our sincere gratitude to the all of the authorities of the heart center who helped to make this research possible.

**Table 1:** Meteorological variables descriptive

**Variable**	**Mean (SD)**	**Range**	**Median**
Wind speed	4.62 (2.36)	0–20	4.0
Wind speed (18 PM)	1.23 (1.71)	0–18	0.0
Wind speed (12 MD)	2.22 (1.67)	0–8	2.0
Wind speed (6 AM)	0.72 (1.26)	0–8	0.0
Daily evaporation	2.79 (2.27)	0–9.6	2.2
Daily rain	1.62 (5.68)	0–69.9	0.0
Daily relative humidity (18 pm)	79.57 (11.75)	26–98	82.0
Daily Relative humidity (12 md)	66.15 (15.98)	26–100	64.0
Daily Relative humidity (6 am)	89.42 (9.14)	30–100	92.0
Daily average relative humidity	78.03 (9.39)	47.5–98.5	78.0
Daily maximum relative humidity	95.11 (4.33)	72–100	97.0
Daily minimum relative humidity	60.95 (16.65)	23–97	60.0
Daily average temperature	16.71 (7.06)	5.5–33.3	15.0
Maximum daily temperature	21.36 (7.99)	7.4–42.6	19.6

**Table 2:** Comparison of ACS by month adjusted for age, meteorological variables and sex

**Reference**	**Month**	**Mean Difference (Reference-Month)**	**95% Confidence Interval for Difference**		**Bonferroni corrected ** ***P*** ** value**
			**Lower**	**Upper**	
Month 1	2	58	-6.1	122.1	0.152
	3	-188	-276.4	-99.6	< 0.001
	4	-325	-428.1	-221.9	< 0.001
	5	-87	-198.3	24.3	0.561
	6	67	-29.3	163.3	1.000
	7	66	-34.7	166.7	1.000
	8	58	-44.8	160.8	1.000
	9	65	-29.7	159.7	1.000
	10	74	-6.0	154.0	0.122
	11	54	-13.1	121.1	0.445
	12	60	-3.3	123.3	0.092
Month 2	3	-246	-333.0	-159.0	< 0.001
	4	-383	-477.9	-288.1	< 0.001
	5	-145	-235.3	-54.7	< 0.001
	6	9	-64.7	82.7	1.000
	7	8	-70.2	86.2	1.000
	8	0	-80.2	80.2	1.000
	9	7	-65.9	79.9	1.000
	10	16	-43.0	75.0	1.000
	11	-4	-56.4	48.4	1.000
	12	2	-47.8	51.8	1.000
Month 3	4	-137	-257.1	-16.9	0.008
	5	101	-31.9	233.9	0.690
	6	255	133.6	376.4	< 0.001
	7	254	128.1	379.9	< 0.001
	8	246	117.1	374.9	< 0.001
	9	253	131.7	374.3	< 0.001
	10	262	154.8	369.2	< 0.001
	11	242	148.2	335.8	< 0.001
	12	248	159.5	336.5	< 0.001
Month 4	5	238	116.7	359.3	< 0.001
	6	392	281.4	502.6	< 0.001
	7	391	277.9	504.1	< 0.001
	8	383	269.9	496.1	< 0.001
	9	390	281.9	498.1	< 0.001
	10	399	299.9	498.1	< 0.001
	11	379	282.8	475.2	< 0.001
	12	385	290.1	479.9	< 0.001
Month 5	6	154	87.2	220.8	< 0.001
	7	153	84.0	222.0	< 0.001
	8	145	75.9	214.1	< 0.001
	9	152	81.7	222.3	< 0.001
	10	161	87.5	234.5	< 0.001
	11	141	53.4	228.6	< 0.001
	12	147	56.4	237.6	< 0.001
Month 6	7	-1	-50.8	48.8	1.000
	8	-9	-59.5	41.5	1.000
	9	-2	-52.2	48.2	1.000
	10	7	-46.8	60.8	1.000
	11	-13	-82.4	56.4	1.000
	12	-7	-80.3	66.3	1.000
Month 7	8	-8	-58.8	42.8	1.000
	9	-1	-51.5	49.5	1.000
	10	8	-48.1	64.1	1.000
	11	-12	-85.4	61.4	1.000
	12	-6	-83.4	71.4	1.000
Month 8	9	7	-43.2	57.2	1.000
	10	16	-39.9	71.9	1.000
	11	-4	-79.5	71.5	1.000
	12	2	-77.4	81.4	1.000
Month 9	10	9	-42.3	60.3	1.000
	11	-11	-78.7	56.7	1.000
	12	-5	-76.5	66.5	1.000
Month 10	11	-20	-76.1	36.1	1.000
	12	-14	-72.0	44.0	1.000
Month 11	12	6	-44.8	56.8	1.000

General Linear Model performed.

**Table 3:** Predictors for acute coronary syndrome

**Variable**	**IRR (95% CI)**	***P *** **value**
**All subjects**		
Male mean (SD): 6.13 (5.70)	1.03 (0.94 to 1.12)	0.598
Female mean (SD): 5.70 (5.10)	1.0	
Age	1.001 (0.997 to 1.004)	0.749
Wind speed (18 PM)	1.051 (1.019 to 1.083)	0.001
Wind speed (12 MD)	0.982 (0.953 to 1.011)	0.220
Wind speed (6 AM)	1.009 (0.970 to 1.050)	0.653
Daily evaporation	1.039 (1.003 to 1.077)	0.032
Daily rain	0.992 (0.985 to 1.00)	0.060
Daily relative humidity (18 PM)	0.985 (0.978 to 0.992)	< 0.001
Daily Relative humidity (12 MD)	0.998 (0.990 to 1.007)	0.683
Daily maximum relative humidity	1.036 (1.023 to 1.050)	< 0.001
Daily minimum relative humidity	1.013 (1.004 to 1.022)	0.003
Maximum daily temperature	1.015 (0.997 to 1.034)	0.100
Minimum daily temperature	0.942 (0.927 to 0.958)	< 0.001
**Males**		
Age	1.002 (0.997 to 1.007)	0.474
Wind speed (18PM)	1.039 (0.997 to 1.083)	0.067
Wind speed (12MD)	0.977 (0.937 to 1.020)	0.291
Wind speed (6AM)	1.015 (0.960 to 1.073)	0.612
Daily evaporation	1.056 (1.003 to 1.112)	0.039
Daily rain	0.991 (0.980 to 1.003)	0.133
Daily relative humidity (18PM)	0.990 (0.981 to 1.000)	0.060
Daily Relative humidity (12MD)	0.998 (0.987 to 1.010)	0.786
Daily maximum relative humidity	1.035 (1.016 to 1.054)	< 0.001
Daily minimum relative humidity	1.009 (0.997 to 1.022)	0.144
Maximum daily temperature	1.011 (0.984 to 1.037)	0.430
Minimum daily temperature	0.945 (0.921 to 0.968)	< 0.001
**Females**		
Age	0.999 (0.995 to 1.004)	0.756
Wind speed (18PM)	1.065 (1.019 to 1.114)	0.006
Wind speed (12MD)	0.986 (0.947 to 1.028)	0.508
Wind speed (6AM)	0.998 (0.944 to 1.050)	0.941
Daily evaporation	1.023 (0.975 to 1.055)	0.356
Daily rain	0.993 (0.983 to 1.004)	0.220
Daily relative humidity (18PM)	0.978 (0.968 to 0.988)	< 0.001
Daily Relative humidity (12MD)	0.999 (0.987 to 1.011)	0.849
Daily maximum relative humidity	1.038 (1.020 to 1.057)	< 0.001
Daily minimum relative humidity	1.017 (1.005 to 1.030)	0.006
Maximum daily temperature	1.021 (0.996 to 1.048)	0.106
Minimum daily temperature	0.939 (0.917 to 0.961)	< 0.001

Negative Binomial regression performed. Abbreviations: IRR, incident relative risk; CI, confidence interval.

## References

[1] Sharif Nia H, Sivarajan-Froelicher E, Haghdoost AA, Moosazadeh M, Huak-Chan Y, Farsavian AA (2018). The estimate of average age at the onset of acute myocardial infarction in Iran: a systematic review and meta-analysis study. ARYA Atheroscler.

[2] Sharif Nia H, Haghdoost AA, Nazari R, Bahrami N, Soleimani MA, Pormand K (2013). Relationship of risk factors and ST segment changes with symptoms of acute coronary syndrome. Koomesh.

[3] Honda T, Fujimoto K, Miyao Y (2016). Influence of weather conditions on the frequent onset of acute myocardial infarction. J Cardiol.

[4] Moschos N, Christoforaki M, Antonatos P (2004). Seasonal distribution of acute myocardial infarction and its relation to acute infections in a mild climate. Int J Cardiol.

[5] Sen T, Astarcioglu MA, Asarcikli LD, Kilit C, Kafes H, Parspur A (2016). The effects of air pollution and weather conditions on the incidence of acute myocardial infarction. Am J Emerg Med.

[6] Kriszbacher I, Bodis J, Csoboth I, Boncz I (2009). The occurrence of acute myocardial infarction in relation to weather conditions. Int J Cardiol.

[7] Goggins WB, Chan EY, Yang CY (2013). Weather, pollution, and acute myocardial infarction in Hong Kong and Taiwan. Int J Cardiol.

[8] Patel NJ, Pant S, Deshmukh AJ, Nalluri N, Badheka AO, Shah N (2014). Seasonal variation of acute myocardial infarction related hospitalizations in the United States: perspective over the last decade. Int J Cardiol.

[9] Ogbebor O, Odugbemi B, Maheswaran R, Patel K (2018). Seasonal variation in mortality secondary to acute myocardial infarction in England and Wales: a secondary data analysis. BMJ Open.

[10] Culic V (2006). Seasonal distribution of acute myocardial infarction: a need for a broader perspective. Int J Cardiol.

[11] Radisauskas R, Bernotiene G, Baceviciene M, Ustinaviciene R, Kirvaitiene J, Kranciukaite-Butylkiniene D (2014). Trends of myocardial infarction morbidity and its associations with weather conditions. Medicina (Kaunas).

[12] Madrigano J, Mittleman MA, Baccarelli A, Goldberg R, Melly S, von Klot S (2013). Temperature, myocardial infarction, and mortality: effect modification by individual- and area-level characteristics. Epidemiology.

[13] Messner T, Lundberg V, Wikstrom B (2002). A temperature rise is associated with an increase in the number of acute myocardial infarctions in the subarctic area. Int J Circumpolar Health.

[14] Morabito M, Modesti PA, Cecchi L, Crisci A, Orlandini S, Maracchi G (2005). Relationships between weather and myocardial infarction: a biometeorological approach. Int J Cardiol.

[15] Loomba RS (2015). Seasonal variation in paroxysmal atrial fibrillation: A systematic review. J Atr Fibrillation.

[16] Rumana N, Kita Y, Turin TC, Murakami Y, Sugihara H, Morita Y (2008). Seasonal pattern of incidence and case fatality of acute myocardial infarction in a Japanese population (from the Takashima AMI Registry, 1988 to 2003). Am J Cardiol.

[17] Spencer FA, Goldberg RJ, Becker RC, Gore JM (1998). Seasonal distribution of acute myocardial infarction in the second National Registry of Myocardial Infarction. J Am Coll Cardiol.

[18] Gonzalez Hernandez E, Cabades O’Callaghan A, Cebrian Domenech J, Lopez Merino V, Sanjuan Manez R, Echanove Errazti I (2004). Seasonal variations in admissions for acute myocardial infarction. The PRIMVAC study. Rev Esp Cardiol.

[19] Akioka H, Yufu K, Teshima Y, Kawano K, Ishii Y, Abe I (2019). Seasonal variations of weather conditions on acute myocardial infarction onset: Oita AMI Registry. Heart Vessels.

[20] Fares A (2013). Winter cardiovascular diseases phenomenon. N Am J Med Sci.

[21] Lin GM, Li YH, Lin CL, Wang JH, Han CL (2013). Seasonal variation in cardiac death of patients with angiographic coronary artery disease from the ET-CHD registry, 1997-2006. Int J Cardiol.

[22] Grant WB, Bhattoa HP, Boucher BJ (2017). Seasonal variations of US mortality rates: Roles of solar ultraviolet-B doses, vitamin D, gene exp ression, and infections. J Steroid Biochem Mol Biol.

[23] Abrignani MG, Corrao S, Biondo GB, Renda N, Braschi A, Novo G (2009). Influence of climatic variables on acute myocardial infarction hospital admissions. Int J Cardiol.

[24] Goudarzian AH, Sharif Nia H, Jafari H, Jamali S, Badiee M, Sayemi Z (2016). Inpatient satisfaction with health system transformation project in mazandaran educational hospitals, Iran. Journal of Mazandaran University of Medical Sciences.

[25] Spielberg C, Falkenhahn D, Willich SN, Wegscheider K, Voller H (1996). Circadian, day-of-week, and seasonal variability in myocardial infarction: comparison between working and retired patients. Am Heart J.

[26] Wang H, Kakehashi M, Matsumura M, Eboshida A (2007). Association between occurrence of acute myocardial infarction and meteorological factors. J Cardiol.

[27] Wang H, Matsumura M, Kakehashi M, Eboshida A (2005). Seasonal variations and the effect of atmospheric temperature on the incidence of coronary heart disease in Hiroshima, Japan. J Health Sci Hiroshima Univ.

[28] Fischer T, Lundbye-Christensen S, Johnsen SP, Schonheyder HC, Sorensen HT (2004). Secular trends and seasonality in first-time hospitalization for acute myocardial infarction--a Danish population-based study. Int J Cardiol.

[29] Mahmoud KD, Lennon RJ, Ting HH, Rihal CS, Holmes DR Jr (2011). Circadian variation in coronary stent thrombosis. JACC Cardiovasc Interv.

[30] Isik T, Ayhan E, Uyarel H, Akkaya E, Ergelen M, Cicek G (2013). Circadian, weekly, and seasonal variation in early stent thrombosis patients who previously underwent primary percutaneous intervention with ST elevation myocardial infarction. Clin Appl Thromb Hemost.

[31] Amiya S, Nuruki N, Tanaka Y, Tofuku K, Fukuoka Y, Sata N (2009). Relationship between weather and onset of acute myocardial infarction: can days of frequent onset be predicted?. J Cardiol.

[32] Li QB, Sheng L, He Y (1997). The effect of climatic factors on the onset of acute myocardial infarction. Zhonghua Hu Li Za Zhi.

[33] Matthews CE, Freedson PS, Hebert JR, Stanek EJ 3rd, Merriam PA, Rosal MC (2001). Seasonal variation in household, occupational, and leisure time physical activity: longitudinal analyses from the seasonal variation of blood cholesterol study. Am J Epidemiol.

[34] Khaw KT (1995). Temperature and cardiovascular mortality. Lancet.

[35] Meier CR, Jick SS, Derby LE, Vasilakis C, Jick H (1998). Acute respiratory-tract infections and risk of first-time acute myocardial infarction. Lancet.

[36] Sunyer J, Ballester F, Tertre AL, Atkinson R, Ayres JG, Forastiere F (2003). The association of daily sulfur dioxide air pollution levels with hospital admissions for cardiovascular diseases in Europe (The Aphea-II study). Eur Heart J.

[37] Isik T, Ayhan E, Uyarel H, Gunaydin ZY, Bektas O, Karagoz A (2015). The Relation between Seasonal Variation and Mortality in Patients with ST Elevation Myocardial Infraction. Acta Med Mediterr.

[38] Stewart S, Keates AK, Redfern A, McMurray JJV (2017). Seasonal variations in cardiovascular disease. Nat Rev Cardiol.

[39] Goerre S, Egli C, Gerber S, Defila C, Minder C, Richner H (2007). Impact of weather and climate on the incidence of acute coronary syndromes. Int J Cardiol.

[40] Aylin P, Morris S, Wakefield J, Grossinho A, Jarup L, Elliott P (2001). Temperature, housing, deprivation and their relationship to excess winter mortality in Great Britain, 1986-1996. Int J Epidemiol.

[41] Messner T, Lundberg V, Wikstrom B (2003). The Arctic Oscillation and incidence of acute myocardial infarction. J Intern Med.

[42] Lee JH, Chae SC, Yang DH, Park HS, Cho Y, Jun JE (2010). Influence of weather on daily hospital admissions for acute myocardial infarction (from the Korea Acute Myocardial Infarction Registry). Int J Cardiol.

[43] Chang CL, Shipley M, Marmot M, Poulter N (2004). Lower ambient temperature was associated with an increased risk of hospitalization for stroke and acute myocardial infarction in young women. J Clin Epidemiol.

[44] Panagiotakos DB, Chrysohoou C, Pitsavos C, Nastos P, Anadiotis A, Tentolouris C (2004). Climatological variations in daily hospital admissions for acute coronary syndromes. Int J Cardiol.

[45] Ministry of Health, Labour and Welfare. Vital statistics of Japan. Available from: https://www.mhlw.go.jp/english/database/db-hw/dl/81-1a2en.pdf. Accessed 2016.

[46] Danet S, Richard F, Montaye M, Beauchant S, Lemaire B, Graux C (1999). Unhealthy effects of atmospheric temperature and pressure on the occurrence of myocardial infarction and coronary deaths. A 10-year survey: the Lille-World Health Organization MONICA project (Monitoring trends and determinants in cardiovascular disease). Circulation.

[47] Ku CS, Yang CY, Lee WJ, Chiang HT, Liu CP, Lin SL (1998). Absence of a seasonal variation in myocardial infarction onset in a region without temperature extremes. Cardiology.

[48] Kysely J, Pokorna L, Kyncl J, Kriz B (2009). Excess cardiovascular mortality associated with cold spells in the Czech Republic. BMC Public Health.

[49] Nayha S (2005). Environmental temperature and mortality. Int J Circumpolar Health.

[50] Gutierrez Loyola A, Druyet Castillo D, Oramas Dominguez I, Veliz Martinez PL (2010). Infarto de miocardio agudo en Cuba. Situación actual. Rev Cub Med Int Emerg.

[51] Rivero A, Bolufe J, Ortiz PL, Rodriguez Y, Reyes MC (2015). Influence of climate variability on acute myocardial infarction mortality in Havana, 2001-2012. MEDICC Rev.

[52] Hodzic E, Perla S, Iglica A, Vucijak M (2018). Seasonal Incidence of Acute Coronary Syndrome and Its Features. Mater Sociomed.

[53] Saligheh M, Bayat A, Behboudi H, Zakeri A, Jamali F (2015). Spatial dispersion of climatic factors in North and central basin of Iran using statistical models. Journal of Geographical Sciences.

[54] Daheshvar T, Danehkar A, Ale Sheikh AA, Ahmadian R (2013). Identifying and Locating the Suitable Urban Development Spots with Application Criteria of Ecosystem (Case Study: Mahmood Abad Township, Mazandaran Province). Town and Country Planning.

